# Decoding γδ T cell anticancer therapies: integrating CRISPR screens with tumor organoids

**DOI:** 10.1038/s41392-023-01678-z

**Published:** 2023-11-13

**Authors:** Jian Zhou, Min Wu, Gen Yang

**Affiliations:** 1 Wenzhou Institute, University of Chinese Academy of Sciences, Wenzhou, 325000 Zhejiang China; 2 School of Physics, Peking University, 100871 Beijing, China

**Keywords:** Preclinical research, Tumour immunology, Cancer therapy, Translational research, Cancer therapy

**Fig. 1:**
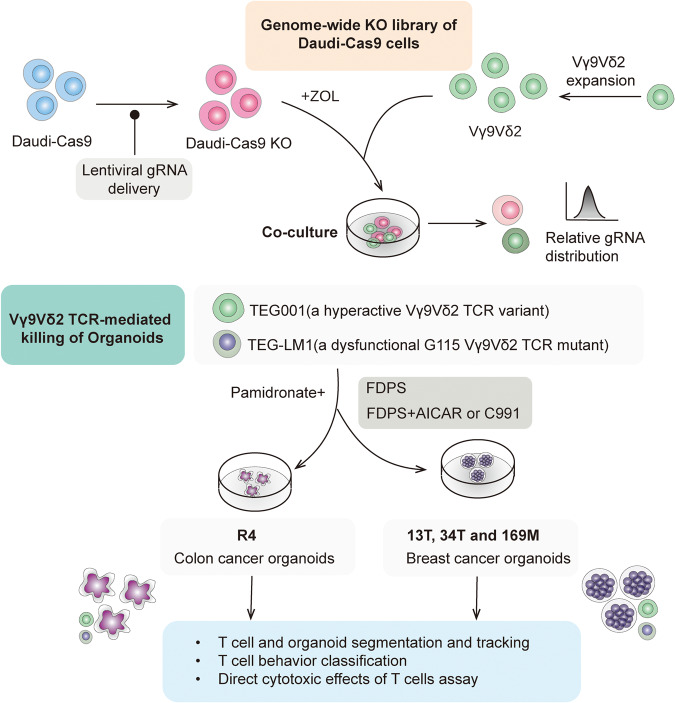
Vγ9Vδ2 T cell genome-wide Cas-9 Screens and TCR-mediated Killing of Cancer cells. Vγ9Vδ2 T cell coculture screen with a genome-wide KO library of Daudi-Cas9 cells, and TCR-mediated killing of breast and colon cancer organoids. ZOL (Zoledronic acid), FDPS (a farnesyl diphosphate synthase inhibitor akin to zoledronic acid), TEG001 (a hyperactive Vγ9Vδ2 TCR variant), TEG-LM1 (a dysfunctional G115 Vγ9Vδ2 TCR mutant). AICAR (a molecule that signals low cellular energy and modulates energy functions), C991 (another regulator of cellular energy dynamics)

In a recent publication in *Nature*,^1^ Mamedov and colleagues identified pathways that modulate γδ T cell killing and BTN3A cellular expression through integrating genome-wide CRISPR screens and tumor organoid culture, deepening our comprehension of γδ T cell stress surveillance and proposing novel pathways to boost γδ T cell’s anticancer functions (Fig. 1).

T cells coordinate immune responses to diverse pathogens and neoplasms via their specific αβ or γδ T cell receptors (TCRs). The successes of several immunotherapies hinge on the sophisticated insight into receptor-ligand interplay, such as the precise dynamics of TCR–MHC engagement and the strategic inhibition of checkpoints like PD1-PDL1.^2^ Unlike αβ counterparts, γδ T cells recognize infections and tumors without relying on traditional HLA interactions, focusing on pervasive cellular alterations. This unique ability bridges rapid innate detection with enduring immunological memory, seamlessly integrating both innate and adaptive immunity.^2^ Considering their prospective tumor responsiveness, it is imperative to elucidate novel tumor-reactive γδ TCR variants to accurately target tumor-associated antigens. Additionally, Vγ9Vδ2 positive T cells, a subset of γδ T cells, distinctively recognize phosphoantigens, critically mediating tumor surveillance and pathogenic responses, whereas Vγ9Vδ2 negative T cells exhibit tissue-specific residency and contribute to diverse immune functions such as wound healing and tissue regeneration. A detailed understanding of the Vγ9Vδ2 TCR’s diverse domains, in relation to ligands and epigenetic modifiers, is essential, as is a deeper grasp of the interplay between γδ T cell subsets and their transformed target cells.^1,2^

The antitumor efficacy of γδ T cells primarily stems from their production of interferon γ (IFNγ) and tumor necrosis factor (TNF). Recent studies have highlighted the role of IL-17-producing γδ T cells, particularly in tandem with immunogenic cell death-inducing chemotherapy agents.^3^ Within tumor environments, the origins of the active γδ T cells—whether infiltrating from circulation or expanding as resident cells within the affected tissue—remain under investigation. However, marked expansion of tissue-resident lymphocytes, inclusive of γδ T cells, has been reported in early spontaneous cancer lesions in mammary (MMTV-PyMT) and prostate (Tramp) mouse models.^2,4^ The γδ TCR repertoire is versatile, recognizing diverse tumor types. Their non-reliance on antigen-presenting molecules positions them as potential candidates for both autologous and allogeneic therapeutic strategies. These expansive properties set specific γδ TCR clones apart from the more specialized αβTCRs of conventional αβ T cells. Studies suggest that γδ T cells are capable of recognizing ubiquitous stress markers present in transformed or distressed cells through both TCR-dependent and TCR-independent pathways.^1^

γδ T cells inherently have the proficiency to identify preserved cellular stress markers recurrent in transformed cells. However, The precise molecular and cellular processes driving stress-induced interactions between γδ T cells and their anomalously modified target cells have yet to be comprehensively elucidated. The Vγ9Vδ2 T cells, which represent the most predominant subset of human γδ T cells, discern a protein complex encompassing butyrophilin 2A1 (BTN2A1) and BTN3A1.^1^ Notably, the BTN2A1-3A1-3A2 surface complex activates upon intracellular phosphoantigen binding from the mevalonate pathway to BTN3A1, enabling BTN2A1 engagement with Vγ9Vδ2 TCRs. While conventional models focus on phosphoantigen abundance as key for Vγ9Vδ2 T cell interactions, Mamedov et al. highlight the modulation of BTN2A1 and BTN3A surface expressions, influenced by cellular metabolic pathways.^1^ This modulation signals Vγ9Vδ2 T cells about changes or stress in target cells, providing a comprehensive understanding of BTN3A regulation by varied metabolic pathways.

Mamedov et al. detailed how AMP-activated protein kinase (AMPK) modulates BTN2A1 and BTN3A expression in energetically stressed cells, revealing a crucial stress-regulated pathway in the interaction between γδ T cells and cancer cells. AMPK activation enhances the susceptibility of these cells to Vγ9Vδ2 TCR-expressing T cell cytotoxicity. In phosphoantigen-absent conditions, BTN3A1 appears to disrupt αβ T cell activity by altering the immunological synapse. Elevated BTN3A1 expression, driven by AMPK, might therefore possess immunosuppressive effects, especially when phosphoantigen levels are low or anti-BTN3A antibodies are absent.^1^ Interestingly, the therapeutic strategy is under clinical evaluation. Collectively, these insights illuminate avenues in γδ T Cell-Based Anticancer therapeutic approaches.

Mamedov and his team investigated Vγ9Vδ2 TCR-mediated cytotoxicity in patient-derived breast and colon cancer organoids (Fig. 1). These organoids underwent treatment with pamidronate, a farnesyl diphosphate synthase(FDPS) inhibitor akin to zoledronic acid(ZOL), either individually or in combination with AICAR, a molecule that signals low cellular energy and modulates energy functions, or with C991, another regulator of cellular energy dynamics. Subsequently, the organoids were co-cultured with TEG cells. These cells are αβ T cells bioengineered to display specific Vγ9Vδ2 TCR clones, in an environment enriched with pamidronate. A comparative analysis was conducted on the cytotoxicity between TEG001 (a hyperactive Vγ9Vδ2 TCR variant) and TEG-LM1 (a dysfunctional G115 Vγ9Vδ2 TCR mutant). Upon AMPK agonist treatment, tumor organoids and Daudi cells showed enhanced susceptibility to TEG001 cytotoxicity. Prior research identified varying sensitivities: 13T and 169M breast cancer variants were notably vulnerable to TEG001, while 34T was resistant. Remarkably, post-treatment, 34T became as susceptible as 169M and 13T, both of which displayed even increased sensitivity. These results emphasize AMPK activation’s profound influence on cancer cell interactions with Vγ9Vδ2 TCR.In light of the ongoing TEG001-focused clinical trial (NTR6541), this research illuminates the potential of integrating AMPK activation into therapeutic strategies.^1,5^

The immune system meticulously maintains cellular and tissue balance, acting when abnormalities exceed usual thresholds. Mamedov et al. describe a mechanism by which human γδ T cells intensely monitor cells. They found that BTN3A expression is influenced by interferon-responsive pathways and metabolic stress indicators. A hallmark of cancer cells is their metabolic shift favoring aerobic glycolysis (Warburg effect) over oxidative phosphorylation (OXPHOS) for ATP, making ATP production less efficient. The tumor environment often exacerbates this by reducing OXPHOS activity due to hypoxia. Mamedov and team point out that for optimal Vγ9Vδ2 TCR activation, two conditions must be met: increased mevalonate pathway phosphoantigens and strong BTN2A1–3A1–3A2 complex expression. This implies γδ T cells can detect cells with a hyperactive mevalonate pathway and weakened OXPHOS. Through their research, Mamedov et al. highlight how Vγ9Vδ2 T cells interact with stressed cancer cells. Their research sheds light on the promising avenue of incorporating AMPK activation within therapeutic regimes.^1^

In sum, Mamedov and colleagues using genome-wide CRISPR screens and organoid verification unveil novel avenues for augmenting Vγ9Vδ2 T cell functionality in oncological patients, thereby potentiating a more efficacious activation against cancer cells under metabolic stress conditions. Utilizing tumor organoids to evaluate the direct cytotoxic effects of T cells, they have provided an invaluable methodological reference for subsequent research and expanded the application horizons of tumor organoids in the field of cancer therapy.^2,5^ Only with such profound insights can we anticipate the realization of the immense potential inherent to γδ T cell-centric therapies.
